# Cost-effectiveness analysis of an outreach model of Hepatitis C Virus (HCV) assessment to facilitate HCV treatment in primary care

**DOI:** 10.1371/journal.pone.0234577

**Published:** 2020-06-17

**Authors:** David Brain, Jonathan Mitchell, James O’Beirne

**Affiliations:** 1 Australian Centre for Health Services Innovation, Melbourne, Australia; 2 School of Public Health and Social Work, Queensland University of Technology, Brisbane, Australia; 3 Sunshine Coast University Hospital, Britinya, Australia; 4 Sunshine Coast Health Institute, University of the Sunshine Coast, Britinya, Australia; Middle East Liver Diseases (MELD) Center, ISLAMIC REPUBLIC OF IRAN

## Abstract

The effects of hepatitis C virus (HCV), such as morbidity and mortality associated with cirrhosis and liver cancer, is a major public health issue in Australia. Highly effective treatment has recently been made available to all Australians living with HCV. A decision-analytic model was developed to evaluate the cost-effectiveness of the hepatology partnership, compared to usual care. A Markov model was chosen, as it is state-based and able to include recursive events, which accurately reflects the natural history of the chronic and repetitive nature of HCV. Cost-effectiveness of the new model of care is indicated by the incremental cost-effectiveness ratio (ICER), where the mean change to costs associated with the new model of care is divided by the mean change in quality adjusted life-years (QALYs). Ten thousand iterations of the model were run, with the majority (73%) of ICERs representing cost-savings. In comparison to usual care, the intervention improves health outcomes (22.38 QALYs gained) and reduces costs by $42,122 per patient. When compared to usual care, a partnership approach to management of HCV is cost-effective and good value for money, even when key model parameters are changed in scenario analyses. Reduction in costs is driven by improved efficiency of the new model of care, where more patients are treated in a timely manner, away from the expensive tertiary setting. From an economic perspective, a reduction in hospital-based care is a positive outcome and represents a good investment for decision-makers who wish to maximise health gain per dollar spent.

## Introduction

Hepatitis C virus (HCV) is a major public health issue in Australia and causes significant morbidity and mortality through the development of cirrhosis and its complications including liver cancer. Since March 2016, highly effective treatment with direct acting anti-viral agents (DAA) have been made available to all Medicare eligible Australians living with HCV [1]. Since this time over 70,000 patients have been treated, however after an initial large expansion of treatment uptake there has been a decline in treatment rates such that as of 2017 approximately 170,000 Australians were still living with HCV [2]. A recent Australian study has shown that Australia could be on track to meet eradication targets if efforts to make treatment widely available are continued [3], however there are issues with falling treatment rates in some Australian jurisdictions. Falling treatment rates are largely due to difficulties in case finding, linkage to care and barriers to access for priority populations such as prisoners and persons who inject drugs (PWID). These problems are especially acute in regional areas where access to HCV care may be limited by distance and resources.

In 2016 there were approximately 300 patients waiting for assessment by the hepatology service in Queensland’s Sunshine Coast region. In addition to the significant burden of HCV in the Sunshine Coast region, where up to 20 referrals were being received on a weekly basis, there was no formal hepatology service in the neighbouring Wide Bay hospital and health service, which has the highest prevalence of HCV in Queensland [4]. Historically, services for the management of HCV rely heavily on the secondary care setting where specialist-driven outpatient clinics are standard practice. This is both difficult from a capacity-management perspective, as hospital services are already stretched in the Australian system, and it is also expensive given the increased cost associated with specialist-led care. The need for a novel, outreach hepatology service that engages primary care physicians, delivers specialist advice in a setting closer to home whilst identifying patients with more advanced disease who will require management in secondary care such as a quality surveillance programme for liver cancer, is significant. Furthermore, costs associated with hospital-based HCV services are not sustainable and the reduction of avoidable face-to-face appointments in the outpatient setting is likely to be of economic benefit. However, this has not been formally quantified with an economic evaluation in the Australian setting. As such, the aim of this study is to evaluate the cost-effectiveness of a novel approach to service delivery for the management and treatment of people living with HCV, compared to usual care.

## Methods

This evaluation follows the guidelines presented in the *Consolidated Health Economic Evaluation Reporting Standards* (CHEERS) checklist [5]. Following this checklist is recommended to ensure consistency and rigour when reporting economic findings. The completed checklist can be found in the S1 File

### Description of service delivery

#### Hepatology partnership model of care

The hepatology partnership aimed to deliver expert assessment of HCV and specialised care using a hub and spoke model. The hub was based at the Sunshine Coast University Hospital, with HCV treatment being delivered to patients in the community by GPs following assessment at community clinics local to patients. During treatment, patients and GPs were supported by the maintenance of close links with the specialist team who led the intervention from secondary care. Referrals to the service were received from GPs, following triage, patients were assessed in the community and then discussed at weekly multidisciplinary team (MDT) meetings, which included consultants and nurses involved in the intervention. Initial appointments were scheduled and undertaken by nurses, with assessment of liver fibrosis using the mobile Fibroscan technology undertaken in the community setting. Patient education was also nurse-led and undertaken in the community setting. Patients requiring treatment for their infection had a drug regimen recommended via the specialist-based hub which was prescribed by the General Practitioner (GP), therefore patients were not required to visit a hospital for treatment unless fibrosis assessment revealed cirrhosis in which case review in secondary care was organised. If treatment failed, the process was repeated, with the exception of the fibrosis assessment, which was only completed once. The service was designed to reduce flow into the hospital setting and provide patients with timely and appropriate care, close to their community. Telehealth and MDT case conferencing was available to ensure a connection between primary and secondary care, and was utilised on an ad-hoc basis.

#### Usual care (historical comparator)

Before the implementation of the intervention, there was a significant reliance on secondary care for the provision of hepatology services. Referrals to the service at the Sunshine Coast hepatology department were received from primary care, where an initial appointment was booked for a specialist-led, hospital outpatient clinic. Following consultation with a hepatology specialist the patient had a fibrosis assessment using the Fibroscan technology, by a hospital-based nurse who also delivered patient education at this appointment. A follow-up appointment was scheduled, requiring another hospital outpatient visit and after treatment had been prescribed and completed, a final hospital outpatient clinic visit was required to check whether cure had been achieved. If treatment failed, the process was repeated, with the exception of the fibrosis assessment, which was only completed once.

### Study design

This evaluation used data that had been collected pre- and post-implementation of the new model of care for patients with HCV. The same data was collected before and after the implementation of the new model care, to allow a pre-/post- analysis. Pre-implementation data was available for 12 months between 2016 and 2017, and was used to determine estimates for usual care. Post-implementation data was collected for 20 months between 2017 and 2018, and was used to determine estimates for the new model of care. Demographic information, treatment history, treatment start date, fibrosis level, method of fibrosis assessment and treatment regimen were collected using the hepatology department’s administrative database. Ethical approval for the study was granted by The Prince Charles Hospital Human Research Ethics Committee, reference number LNR/2018/QPCH/47452. All data was fully anonymised when the research team accessed it.

### Decision-analytic model

**Fig 1:**
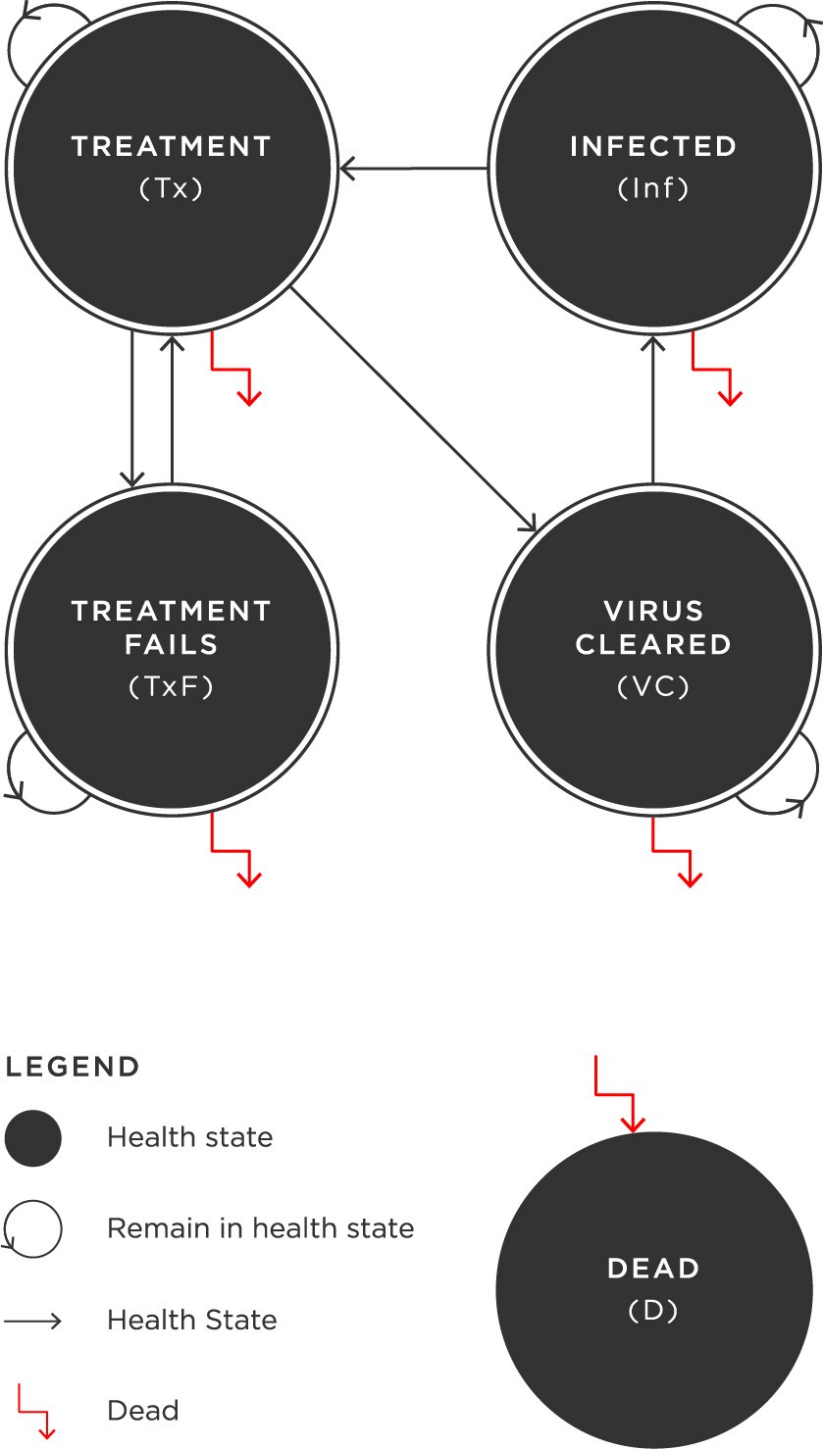
Pictorial representation of the Markov model used for economic evaluation of the hepatology partnership.

A decision-analytic model was developed to evaluate the cost-effectiveness of the hepatology partnership, compared to usual care. A Markov model was chosen, as it is state-based and able to include recursive events, which accurately reflects the natural history of the chronic and repetitive nature of HCV [6]. A pictorial representation of the model is shown in Fig 1. The model depicts the possible movement of patients through its health states over time, with each health state having a cost and a health utility weight attached to time spent in that state. This structure provides the framework for the evaluation and is used to estimate the costs and health outcomes associated with the differing approaches to service provision. The model contains five health states; ‘infected’, ‘receiving treatment’, ‘treatment fails’, ‘virus cleared’ and an absorbing state, ‘dead’. For the base case analysis, the model’s cycle length was 1 week and it was run for 52 cycles to complete a 1-year time horizon. The modelled patient cohort was a 70:30 ratio of male and female patients, with an average age of 45 years. This demographic breakdown is in line with similar studies in this specialty, both from Australia and elsewhere in the world, and reflects the average age and gender split of the patient cohort of this study [7,8]. Further information on patient characteristics are shown in Table 1.

**Table 1:** Baseline patient characteristics.

	Usual Care (n = 102)	Intervention (n = 378)
**Age in years, Mean (SD)**	46.17 (12.41)	47.47 (11.56)
**Male (%)**	70	67
**Fibrosis Level (%)**		
**F0**	40.59	7.96
F1	26.73	55.72
F2	21.78	11.44
F3	3.96	13.18
F4	6.93	11.69
**Treatment History**		
Naïve: Experienced (%)	96:4	92:8
Avg. Time to Treatment	46 days	28 days
Assessed with Fibroscan (%)	84.31	94.71
Treatment success (%)	84	88

Because we are comparing the costs and health outcomes of treatment for patients with hepatitis C virus, all patients begin in the ‘infected’ health state, represented by each circle. At each cycle of the model, patients in this state can remain infected, enter the ‘receiving treatment’ state, or die. Patients ‘receiving treatment’ can remain in this state, have their condition improve and enter the ‘virus cleared’ state, enter the ‘treatment fails’ state or die. Those who have a treatment failure can remain in that state, return to the ‘receiving treatment’ state, or die. Capacity to return to treatment is an important, realistic representation of a typical approach to treatment. For patients whose virus has cleared, the majority remain in this state, a small proportion will return to the ‘infected’ health state—representing re-infection, and some will die. Patients being treated under the hepatology partnership have different transition probabilities associated with their movement through the model, compared to those receiving usual care. The model was constructed and analysed in Microsoft Excel 2010.

### Input data for the model

Movement between health states is based on transition probabilities, which were estimated from various sources shown in in Table 2. The cost and health outcomes associated with being in each health state were calculated per cycle and then summed over the 52 cycles of the model. The model was run for a period of 1 year and as such, discounting was not applied to costs or health outcomes [9].

**Table 2:** Input variables for the Markov model.

Variable	Fixed Value	Distribution	Ref
**Transition Probabilities**^+^	Mean (α, β)		
**Usual Care**			
Infected to Treatment	0.25 (α = 25,β = 75)	Beta	Study cohort
Treatment to Treatment Fails	0.05 (α = 5,β = 95)	Beta	Study cohort
Treatment to Virus Cleared	0.84 (α = 84,β = 16)	Beta	Study cohort
Virus Cleared to Infected	0.02 (α = 2,β = 98)	Beta	[7]
Treatment to Dead	0.0016 (α = 312,β = 194711)	Beta	[11]
Treatment Fails to Treatment	0.6 (α = 60,β = 40)	Beta	Assumption
Treatment Fails to Dead	0.0016 (α = 312,β = 194711)	Beta	[11]
Virus Cleared to Dead	0.0016 (α = 312,β = 194711)	Beta	[11]
Infected to Dead	0.0016 (α = 312,β = 194711)	Beta	[11]
**Hepatology Partnership**			
Infected to Treatment	0.5 (α = 50,β = 50)	Beta	Study cohort
Treatment to Treatment Fails	0.034 (α = 9,β = 251)	Beta	Study cohort
Treatment to Virus Cleared	0.88 (α = 229,β = 31)	Beta	Study cohort
Virus Cleared to Infected	0.02 (α = 2,β = 98)	Beta	[7]
Treatment to Dead	0.0016 (α = 312,β = 194711)	Beta	[11]
Treatment Fails to Treatment	0.6 (α = 60,β = 40)	Beta	Assumption
Treatment Fails to Dead	0.0016 (α = 312,β = 194711)	Beta	[11]
Virus Cleared to Dead	0.0016 (α = 312,β = 194711)	Beta	[11]
Infected to Dead	0.0016 (α = 312,β = 194711)	Beta	[11]
**Utility Weights**	Mean (SD)		
Infected	0.66 (0.27)	Beta	[13], Annual
Treatment	0.77 (0.51)	Beta	[13], Annual
Treatment Fails	0.66 (0.27)	Beta	[13], Annual
Virus Cleared	0.85 (0.22)	Beta	[14], Annual
**Costs**			
**Usual Care**			
Infected	$1,389 (α = 1,β = 1128)	Gamma	Study cohort, weekly
Treatment Fails	$555.92 (α = 1,β = 767)	Gamma	Study cohort, weekly
**Hepatology Partnership**			
Infected	$571.40 (α = 1,β = 517)	Gamma	Study cohort, weekly
Treatment Fails	$425.16 (α = 1,β = 416)	Gamma	Study cohort, weekly

^+^Annual probabilities were transformed to weekly probabilities using the formula: tp = 1-(1-tpt)1/t [6].

#### Transition probabilities

Where available, we used data collected from the study to estimate transition probabilities for the model. For transitions that could not be estimated from primary data, such as death from all causes, we used estimates that were published in the literature. For this type of evaluation, it is not uncommon to use a variety of sources to estimate transition probabilities [10]. We used prospectively collected data to estimate the probability of receiving treatment, the probability of a patient achieving cure and the probability of treatment failing, for both groups. To inform estimates for the usual care group, data was collected for 12 months prior to the new model of care. For estimates associated with the intervention, data was collected over a 20-month study period. To estimate the rare probability of re-infection after virus has been cleared, the same rate that was used in a previously published study was used for this model [7]. All-cause mortality was calculated using age and gender specific estimates from the Australian Bureau of Statistics that represent the modelled cohort [11]. Due to the low percentage of patients in our study being cirrhotic, the background risk of hepatocellular carcinoma in this cohort is very low. As such, due to the unlikeliness of death due to HCV, we assumed no difference in all-cause mortality and HCV-related mortality. The model’s cycle length was weekly, meaning that all probabilities were transformed using a standardised formula [6]. The probability of remaining in a health state was simply calculated as 1 minus the sum of the other probabilities associated with leaving that health state [12]. Sources of evidence and values used in the model are summarised in Table 2.

#### Quality of life

Due to a large body of work in the area of HCV quality of life already existing, health utility estimates for use in the model were extracted from the published literature and were not prospectively collected as part of the study. The new drug regimen available for patients living with HCV are widely reported to be safe, with very few adverse events reported in studies from around the world [15,16]. This is supported by the data collected in this project, where there were no serious adverse events reported by patients undergoing treatment, either pre- or post-implementation of the partnership. The literature shows that the new drug regimens prescribed to patients living with HCV are well tolerated and that compliance is generally high, even in vulnerable patient populations, so we have assumed that patient experience of treatment, including patient reported quality of life is largely homogenous [17,18]. Several papers stratify health-related quality of life associated with having HCV according to severity categories, and as such, in our base case we assumed that estimates for ‘moderate’ disease would represent the utility of being ‘infected’ in our model. We also assumed that if treatment failed, and patients restarted their treatment, that health utility would be the same as a first-time infection. In sensitivity analysis, we explored changes to the results if the health utility for the infected health states were varied according to the reported ranges in previously published literature. In comparison to the two infected health states, having the virus cleared was associated with an increased health utility.

#### Costs

Costs associated with each health state were measured and valued in 2018 Australian dollars. We used a previously published method for costing the intervention, which included costs associated with staff, space and the resources required to undertake the intervention [19]. Costs were associated with the ‘infected’ and ‘treatment fails’ health states as it is these health states where patients have contact with clinical care and staff, space and consumable costs are accrued. Staff costs such as those associated with triage, booked appointments and patient education were measured and included for both the new model and usual care using a monthly costing survey. Equipment costs, such as the mobile Fibroscanner, travel costs and other costs such as stationery, maintenance contracts and clinic space hire were also measured using routinely collected finance department data, and valued for both the new model and usual care. There are no costs associated with the ‘virus cleared’, ‘treatment’ or ‘dead’ health states. We did not include the cost of drug regimens for treatment of HCV for two reasons–first, the cost of the regimen is heavily subsidised by the Federal government and is not a cost that is of interest to local decision-makers, as they do not foot the bill for treatment. Secondly, because the purpose of the intervention is to increase uptake of HCV treatment, including drug treatment costs would unfairly disadvantage the intervention in comparison to a less effective model of care, where less people are receiving treatment. We did not measure patient out of pocket costs, and the model does not include personal lost productivity or other societal costs. As such, our costing perspective is best described as a restricted healthcare perspective [20].

#### Model outputs

The main purpose of this evaluation is to estimate the expected value for money of the new model of care for patients with HCV, compared to usual care. This is indicated by the incremental cost-effectiveness ratio (ICER), where the mean change to costs associated with the new model of care is divided by the mean change in quality adjusted life-years (QALYs) [21]. The ICER is compared against a cost-effectiveness threshold, which is assumed to be the willingness to pay (WTP) for an additional QALY, with ICERs that fall under the threshold deemed to be ‘cost-effective’. The threshold for this study is $28,000 (AUD), which is based on the most recently published estimates for the Australian healthcare setting [22].

#### Handling uncertainty

To quantify the impact of uncertainty relating to the model’s inputs on the model outputs, probabilistic sensitivity analysis was undertaken. Ten thousand iterations of the model were undertaken, using Monte Carlo simulations, where each simulation takes a random draw from each parameter’s distribution [10]. Transition probabilities and health utilities were assigned beta distributions, while costs were assigned gamma distributions, which reflects the skew that is usually associated with this type of data [10]. The change to costs and the change to QALYs was recorded for each model simulation, producing 10,000 pairs of incremental costs and effects. However, given that the ratio of two numbers has awkward statistical properties, practical issues with using the ICER for decision-making exist [23]. To simplify this ratio information into a single number, the net monetary benefit (NMB) framework is used. Results are converted from the ICER to a NMB value, through the linear rearrangement of the ICER equation, as follows:

NMB = (WTP threshold x Change in Effects)–Change in Costs

The interpretation of cost-effectiveness becomes particularly simple using the NMB: a positive NMB indicates that a strategy is cost-effective and a negative NMB indicates that a strategy is not cost-effective [24]. Using the net benefit framework gives decision-makers a clear framework for choosing to adopt, or not adopt the intervention being evaluated, without needing an in-depth understanding of the ratio of two numbers.

We also changed key parameter values in the model to reflect plausible scenarios, allowing us to explore aspects of uncertainty in the evaluation and to test the robustness of the model. This also increases the extent of information that is available to decision-makers. We considered six alternate scenarios: (1) where the utility value for the infected health state was lower than in the baseline model; (2) where the probability of cure is the same for both the usual care and intervention group; (3) where the probability of dying from infection in the intervention group is higher than in the baseline model; (4) where the decision-maker’s willingness to pay for health benefits is $0; (5) where the probability of being treated is the same for both the usual care and intervention group; and (6) where the time horizon for the model was extended from one year, to ten years.

## Results

### Deterministic analysis

Without consideration of uncertainty in the model’s parameters, it is estimated that the hepatology partnership is a cost-effective approach to the management of patients living with HCV. For a modelled cohort of 1,000 patients, the average change to costs is $42,122,435 lower if the intervention was undertaken, compared to usual care. In addition, the hepatology partnership is associated with an increase of 22.38 QALYs (see Table 3). The results suggest that on average, the per patient cost of services provided via the hepatology partnership are $42,122 lower than when services were provided under the previous model of care. There appears to be an opportunity for significant, system-wide savings to be made if the new model of care is adopted.

**Table 3:** Deterministic results of the economic model.

Group	Total Costs	Total QALYs	Δ Costs	Δ QALYs	ICER^^^
**Usual Care**	$64,025,656	690.94			
**Hepatology Partnership**	$21,903,221	713.32	-$42,122,435	22.38	Dominant*

^ICER: incremental cost-effectiveness ratio

*Because the intervention has better health outcomes and lower costs in comparison to usual care, it is said to be the *dominant* strategy

### Probabilistic analysis

**Fig 2:**
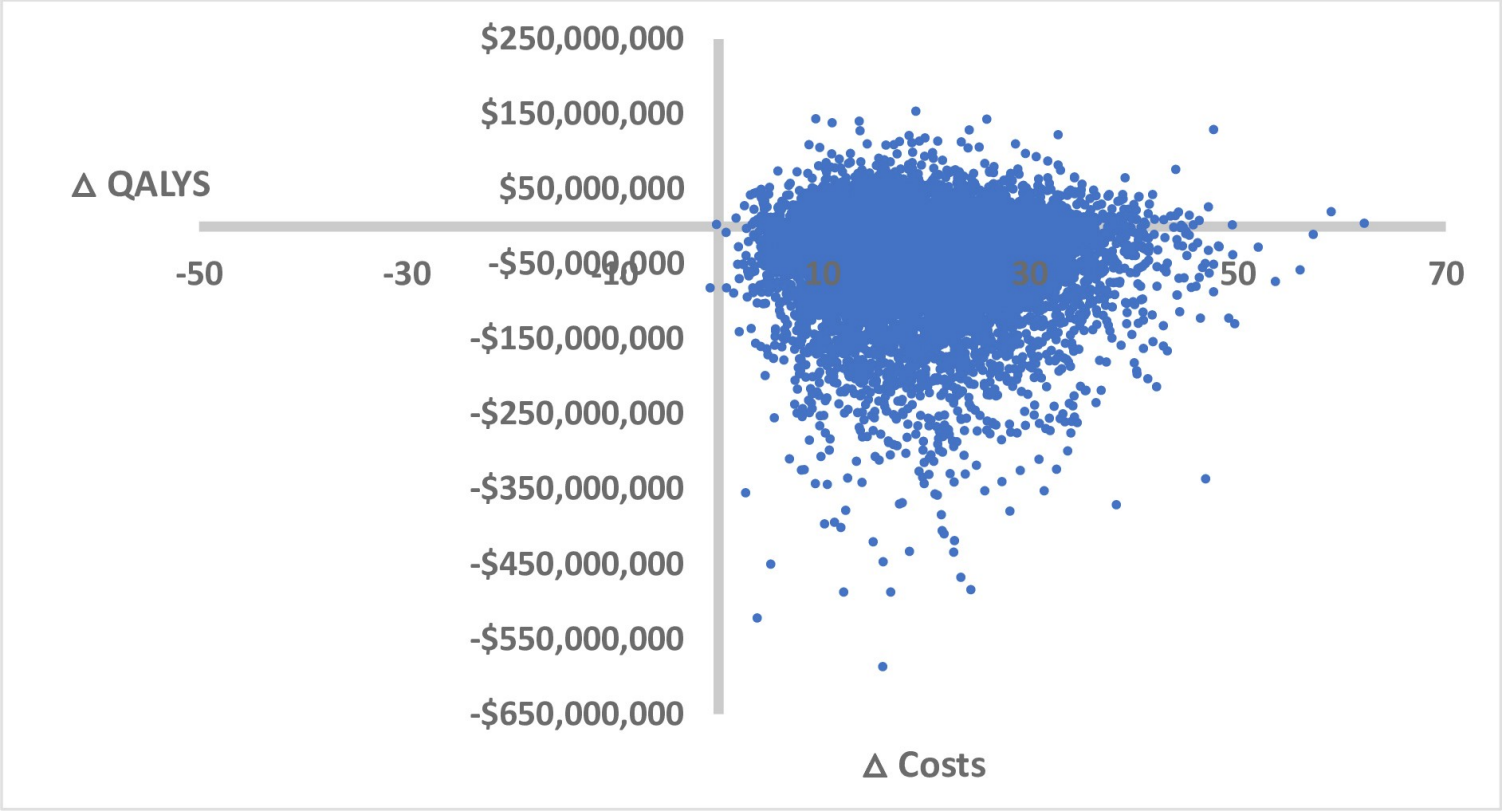
ICER cloud of 10,000 model simulations.

To account for parameter uncertainty, Monte Carlo simulation was undertaken. The results can be seen in Fig 2, which shows the results of running 10,000 simulations of the model. The majority (73%) of ICERs are in the south-east quadrant—the area of the cost-effectiveness plane that represents cost-saving. Put simply, in comparison to usual care, the intervention improves health outcomes and reduces costs. A net monetary benefit (NMB) analysis was also undertaken and provides easily interpreted results, where a positive mean NMB represents a cost-effective intervention and a negative mean NMB represents an intervention that is not cost-effective. At a willingness to pay threshold of $28,000, the mean NMB for the Hepatology Partnership is $42,324,895, which supports the conclusion that the intervention is better value for money when compared with usual care. There is good confidence in the findings, with 75% of the model’s 10,000 simulations indicating that the Hepatology Partnership project is cost-effective. For a decision-maker who is interested in maximising health gain per dollar spent, the hepatology partnership is the optimal strategy for managing patients with HCV.

### Scenario analysis

Uncertainty in other aspects of the evaluation also exists and is explored through scenario analyses. Different scenarios were examined, where key parameters of the model were altered. The modified scenarios reflected plausible situations and test the robustness of the model. Five alternate scenarios were considered: (1) where the utility value for the infected health state was lower than in the baseline model, reflecting the variation in disease severity; (2) where the probability of cure is the same for both the usual care and intervention group, accounting for the possible overestimate of treatment effect in the baseline model; (3) where the probability of dying from infection in the intervention group is higher than in the baseline model, using an estimate from the literature rather than the assumption that risk of death while living with HCV is the same as all-cause mortality; (4) where the decision-maker’s willingness to pay for health benefits is $0, to account for decision-makers who do not want to pay for health benefits; (5) where the probability of being treated is the same for both the usual care and intervention group, to reduce the effect of the shortened time to treatment that was observed in the post-intervention period; and (6) where the time horizon for the model was extended from one year, to ten years, to examine the impact of the new model of care on a longer-term basis. The results of scenario analyses are summarised in Table 4. The Hepatology Partnership was deemed to be the optimal strategy for all scenarios. There was high certainty in these outcomes, with the probability that the Hepatology Partnership was cost-effective being greater than 70% in all tested scenarios. Scenario 6, where the time horizon was extended to 10 years, was the scenario with the most certain decision–it was cost-effective in over 85% of all model simulations. This result shows that the decision to adopt the new model of care would not only be a good short-term choice from an economic perspective, it would also be of benefit for any decision-maker who is concerned with the long-term consequences of adoption.

**Table 4:** Results from probabilistic analysis, including scenario analyses.

Scenario	Mean NMB (Min:Max)	Optimal Strategy	Probability cost-effective
Baseline	$42,324,895 (-$153,684,790:$586,657,601)	Hepatology Partnership	75.48%
Scenario 1	$41,706,615 (-$158,741,738:$513,976,965)	Hepatology Partnership	74.79%
Scenario 2	$42,853,471 (-$180,129,083:$685,842,216)	Hepatology Partnership	75.90%
Scenario 3	$43,087,548 (-$167,339,080:$540,996,152)	Hepatology Partnership	75.28%
Scenario 4	$41,776,443 (-$154,216,110:$586,215,445)	Hepatology Partnership	74.82%
Scenario 5	$31,262,173 (-$225,402,153:$495,092,722)	Hepatology Partnership	70.81%
Scenario 6	$134,179,586 (-$253,898,079:$1,476,532,080)	Hepatology Partnership	86.78%

## Discussion

The aim of this evaluation is to present new evidence about the cost-effectiveness of a partnership approach between primary and secondary care for the treatment and management of HCV in the Australian setting. Our results show that when compared to usual care, a partnership approach is cost-effective and good value for money. Reduction in costs is driven by improved efficiency of the new model of care, where more patients are treated in a timely manner, away from the expensive tertiary setting. From an economic perspective, a reduction in hospital-based care is a positive outcome. Additionally, the partnership model of care is associated with slightly better quality of life outcomes, in comparison to usual care. A reduced rate of re-infection and a longer period of improved quality of life are gains that reduce the economic burden associated with HCV.

Given that the vast majority of patients with HCV do not have advanced fibrosis or cirrhosis and treatment for these individuals is simple and effective there is a need for models of care that allow treatment at scale and pace to achieve the World Health Organization’s goal of HCV elimination by 2030. Basing treatment in secondary or tertiary care is a barrier to access for patients in regional settings and is not suitable for patients who find engagement in secondary care difficult such as PWID or patients who are incarcerated. In Australia, unlike other areas of the world DAA can be prescribed by GPs. This increases the treatment workforce and allows a great opportunity for treatment to take place close to the patient’s home. However, many GPs are not experienced in HCV treatment and therefore may be reluctant to commence therapy. Our treatment model supports GPs to initiate treatment following expert assessment of the patient in the community whilst at the same time identifying those with more advanced disease.

To date, the majority of the Australian literature has focussed on reporting the epidemiology and natural history of HCV [25,26], assessing incidence reduction in specific sub-populations [27] and efficacy of treatment regimens [28,29]. Evaluations from an economic perspective have been conducted and published in the Australian setting but the most recent findings relate to the cost-effectiveness of needle and syringe programs [30,31], and the cost-effectiveness of treatment using new regimens (DAAs) in a specific sub-population of interest—people who inject drugs [32]. Our study provides novel evidence about the cost-effectiveness of a new model of care, specific to the Australian setting, which is designed to provide fast-acting drug treatment, closer to a patient’s home. It should be used to support a change in management practices for patients living with HCV and should be considered for adoption in other jurisdictions that would benefit from a hub and spoke model of care. The results show that for decision-makers who are interested in maximising health gain from their scarce resources, failing to invest in this model of care will mean that cost savings and health outcome gains will be foregone.

Further, improved rates of cure for patients who access treatment in a timely manner reduces the costs associated with seeking treatment and contributes to improvements in health-related quality of life. Patients treated via the new model of care were able to access treatment faster and had a slightly higher rate of achieving cure, compared to patients treated via usual care. Access to timely and local treatment should be advocated, to ensure as many health benefits are achieved as possible. We also believe that the new model of care helps reduce stigma associated with accessing treatment for HCV, because treatment can be accessed outside of hospital. Increased patient acceptability of treatment is an important step in reducing transmission and improving cure rates and should be a focus of future studies in this area.

### Limitations

Our study is not without limitations. The results of the study have been produced using primarily Australian sources and as such, the results are largely intended for use in the Australian setting. However, the study is based on a flexible and easily updated modelling framework, which can cater for inputs from newly identified literature or different healthcare settings to be included, making the results more appropriate for local interpretation in other jurisdictions around the world. This flexibility also means that there is capacity to include other models of care that exist for the management of HCV for comparison to the intervention described in this study.

The results of all modelling studies are somewhat dependent on the assumptions made regarding each parameter’s estimates. In our study, we were unable to estimate the rate with which people recommenced treatment if their first treatment failed, so we assumed that this was 60% in both usual care and under the new model of care. We also assumed that the probability of death whilst infected with HCV was no different to the normal probability of dying as a 45-year-old. For costs associated with the ‘infected’ health state, our calculations are based on treating 8 patients per month under standard care, whereas for the new model of care we have assumed 18 patients were treated per month. The increased efficiency is a direct result of the improvements associated with the new model of care and were informed by the pre- and post-implementation data collection. The same level of relative efficiency improvement may not be observed in other jurisdictions, and will accordingly have an impact on cost outcomes. This should be noted if analysts choose to replicate this study in another jurisdiction. State transitions were validated with clinical opinion before the model was run, but like all models, they do represent a simplification of all clinical possibilities. We make no differentiation between infection severity, patients in our model are either classified as infected—and thus included, or not. As shown in Table 1, we did not have an equal representation of fibrosis levels (F0 –F4) before and after the implementation of the new model of care. This was due to the pre/post study design where all patients accessing care were included in the study rather than focusing on enrolling a matched cohort. Future studies may seek to alter this design, or attempt to determine the effects of a more equal representation of fibrosis level, to see if it impacts the results, but this was not the focus of this study. Finally, we didn’t prospectively collect health-related quality of life data and used estimates from the literature. This is not uncommon for this type of evaluation but does mean that we have assumed relative homogeneity between the population from where literature estimates have been derived, and our hypothetical cohort.

## Conclusion

The focus of this evaluation has been to provide new evidence regarding the economic benefit of a new model of care for the diagnosis and treatment of Hepatitis C Virus in the Australian healthcare setting. Our findings show that cost-savings can be achieved if the provision of services is taken away from the tertiary setting and Hepatologist–GP partnership models are actively pursued. Investment to support a partnership approach would be a cost-effective use of scarce healthcare resources and improve the way that HCV is managed in Australia.

## Supporting information

S1 File Completed CHEERS checklist. (DOCX)
